# Supplementary material for: International medical graduates’ experiences of clinical competency assessment in postgraduate and licensing examinations: A scoping review protocol
Source: PLoS One. 2024 Nov 13;19(11):e0305014. doi: 10.1371/journal.pone.0305014 (PMC11559970; doi:10.1371/journal.pone.0305014)
Supplement: S2 Appendix — Search Strategy for PubMed (National Library of Medicine)—Search conducted on Jan 20th, 2024. (DOCX) [file pone.0305014.s002.docx]

### Appendix 2: Search strategy

### PubMed (National Library of Medicine)

Search conducted on May 10th^th^, 2024.

| **Search** | **Query** | **Records retrieved** |
| --- | --- | --- |
| #1 | "international medical graduate*"[Title/Abstract] OR "foreign medical graduate*"[Title/Abstract] OR "foreign doctor*"[Title/Abstract] OR "foreign trained physician*"[Title/Abstract] OR "foreign trained doctor*"[Title/Abstract] OR "overseas trained doctor*"[Title/Abstract] OR "overseas doctor*"[Title/Abstract] OR "overseas graduate*"[Title/Abstract] OR "Overseas Medical Graduate*"[Title/Abstract] OR "migrant physician*"[Title/Abstract] OR "migrant doctor*"[Title/Abstract] OR "immigrant doctor*"[Title/Abstract] OR "non-EU doctor*"[Title/Abstract] OR "non- EU trained*"[Title/Abstract] OR "non-UK graduate*"[Title/Abstract] OR "non-UK qualified"[Title/Abstract] OR "non-UK trained"[Title/Abstract] OR "non-US graduate*"[Title/Abstract] OR "non-US trained"[Title/Abstract] OR “refugee doctor*”[Title/Abstract] | 2,220 |
| #2 | "foreign medical graduates"[MeSH Terms] | 3631 |
| #3 | ("clinical competenc*"[Title/Abstract] OR "licenc*"[Title/Abstract] OR licens*[Title/Abstract]) OR "accredit*"[Title/Abstract] OR "educational measurement"[Title/Abstract] OR "educational assessment"[Title/Abstract] OR Attainment[Title/Abstract] OR success*[Title/Abstract] OR fail*[Title/Abstract] OR "academic performance"[Title/Abstract]) OR (((Postgraduate[Title/Abstract] OR licenc*[Title/Abstract]) OR licens*[Title/Abstract]) AND (medical[Title/Abstract]) AND (assess*[Title/Abstract] OR exam*[Title/Abstract]))) | 2,764,181 |
| #4 | "clinical competence" OR "educational measurement"[MeSH Terms] | 168,635 |
| #5 | #1 OR #2 | 4619 |
| #6 | #3 OR #4 | 2,903,826 |
| #7 | #5 AND #6 | 1403 |
| #8 | #7 (from 2009 – 2024) | 653 |

This strategy used in PubMed will be revised for use with British Education Index, ERIC, Psych Info, Scopus, and Soc Index
